# Generation of virus‐resistant potato plants by RNA genome targeting

**DOI:** 10.1111/pbi.13102

**Published:** 2019-03-08

**Authors:** Xiaohui Zhan, Fengjuan Zhang, Ziyang Zhong, Ruhao Chen, Yong Wang, Ling Chang, Ralph Bock, Bihua Nie, Jiang Zhang

**Affiliations:** ^1^ State Key Laboratory of Biocatalysis and Enzyme Engineering School of Life Sciences Hubei University Wuhan China; ^2^ Key Laboratory of Potato Biology and Biotechnology Ministry of Agriculture and Rural Affairs National Center for Vegetable Improvement (Central China) Huazhong Agricultural University Wuhan China; ^3^ Max‐Planck‐Institut für Molekulare Pflanzenphysiologie Potsdam‐Golm Germany

**Keywords:** CRISPR/Cas13a, RNA targeting, *Potato virus Y*, virus interference, molecular immunity, virus resistance

## Abstract

CRISPR/Cas systems provide bacteria and archaea with molecular immunity against invading phages and foreign plasmids. The class 2 type VI CRISPR/Cas effector Cas13a is an RNA‐targeting CRISPR effector that provides protection against RNA phages. Here we report the repurposing of CRISPR/Cas13a to protect potato plants from a eukaryotic virus, *Potato virus Y* (PVY). Transgenic potato lines expressing Cas13a/sgRNA (small guide RNA) constructs showed suppressed PVY accumulation and disease symptoms. The levels of viral resistance correlated with the expression levels of the Cas13a/sgRNA construct in the plants. Our data further demonstrate that appropriately designed sgRNAs can specifically interfere with multiple PVY strains, while having no effect on unrelated viruses such as PVA or *Potato virus S*. Our findings provide a novel and highly efficient strategy for engineering crops with resistances to viral diseases.

## Introduction

Potato (*Solanum tuberosum* L.) is one of the world's most important food crops. With nearly 400 million tons produced per year, it ranks fourth after rice, wheat and corn (Halterman *et al*., 2015). However, substantial harvest losses occur due to the plant's susceptibility to a wide range of pathogens, among which *Potato virus Y* (PVY) is one of the most devastating viral pathogens. PVY is a member of the genus *Potyvirus* in the family *Potyviridae*. PVY affects both yield and tuber quality, and can cause crop losses of up to 80% (Quenouille *et al*., 2013). The symptoms induced by PVY include mosaic, mottled and crinkled leaves as well as leaf and vein necrosis (Faurez *et al*., 2012; Karasev and Gray, 2013; Quenouille *et al*., 2013).

The genome of PVY is a single‐stranded, positive‐sense (+) RNA, with a length of ~9.7 kb. The RNA genome is translated into a single large polyprotein of approximately 3061 amino acid residues, and subsequently processed by three virus‐specific proteases into the following mature proteins (viral factors): P1, HC‐Pro, P3, 6K1, CI, 6K2, NIa (VPg plus Pro), NIb (viral replicase) and CP (capsid protein; Quenouille *et al*., 2013). In addition, a small open reading frame (ORF), PIPO, was recently found to be embedded within the P3 region (Chung *et al*., 2008) and shown to be required for efficient viral movement (Wei *et al*., 2010). PVY is prone to high mutation and recombination rates, generating a high level of genetic diversity and a large number of different PVY strains (Davie *et al*., 2017; Karasev and Gray, 2013). Currently, the main PVY strains are PVY^O^, PVY^N^, PVY^NTN^ and PVY^N:O^ (Karasev and Gray, 2013).

Control of PVY and other viruses has been attempted by conventional breeding, transgenic approaches and gene silencing strategies (Chaudhary, 2018). However, its high rates of mutation and recombination allow the virus to evade these strategies, leading to rapid breakdown of resistance. For example, PVY isolates that have overcome the resistance gene *va* of tobacco plants were reported (Janzac *et al*., 2014; Nicolas *et al*., 1997; Takakura *et al*., 2018). Transgenic RNAi against plant viruses has been reported (Pooggin, 2017), but through long‐term co‐evolution, eukaryotic viruses have acquired effective mechanisms of resistance to RNAi. The production of viral suppressors of RNA silencing (VSRs) is a widespread counter‐defense strategy employed (Incarbone and Dunoyer, 2013). For example, VSRs inhibit ARGONAUTE function by precluding target RNA binding to pre‐assembled RISC (Kenesi *et al*., 2017). Another undesired property of RNAi approaches is that constitutive siRNA expression may lead to off‐target effects (Pooggin, 2017). For all these reasons, new strategies for improved broad‐spectrum resistance to multiple PVY strains in potato are urgently needed.

The clustered regularly interspaced short palindromic repeat and associated proteins (CRISPR/Cas) system provides bacteria and archaea with molecular immunity against invading phages and conjugative plasmids (Barrangou and Marraffini, 2014; Marraffini, 2015; Shmakov *et al*., 2017). The CRISPR/Cas system has been widely employed to combat DNA viruses in eukaryotes (Hadidi *et al*., 2016; Mahas and Mahfouz, 2018). By designing appropriate guide RNAs (sgRNAs), CRISPR/Cas9 can cleave the double‐stranded DNA of viral genomes and, in this way, confer resistance to DNA viruses (Ali *et al*., 2015; Ji *et al*., 2015; Zaidi *et al*., 2016). A related system, CRISPR/Cas13a (formerly known as C2c2), was shown to cleave single‐stranded RNA, thus providing defense against invading RNA phages (Abudayyeh *et al*., 2016). The CRISPR/Cas13a system can be engineered to mediate the specific knock‐down of RNA transcripts. This property makes it somewhat comparable to RNA interference, while having the advantage of reduced off‐target effects in both mammalian and plant cells (Abudayyeh *et al*., 2017).

The potential of the CRISPR/Cas13a system to interfere with viral RNA replication has recently been tested with the *Turnip mosaic virus* (TuMV) in the model plant *Nicotiana benthamiana* in transient transformation assays using a tobacco virus for sgRNA delivery. The data suggest that a reduction in the viral load can be achieved (Aman *et al*., 2018). Here, we have investigated the potential of the CRISPR/Cas13a system to confer stable resistance to an important viral disease in a major crop. By designing sgRNAs against conserved coding regions of three different PVY strains, we demonstrate that the CRISPR/Cas13a system can be engineered to confer broad‐spectrum resistance of transgenic potato plants against multiple PVY strains. We also show that the anti‐PVY CRISPR/Cas13a specifically targets PVY genomes, but not other viral genomes (PVA and PVS). Importantly, the efficiency of PVY inhibition was positively correlated with the Cas13a/sgRNA expression levels. Our study suggests that the CRISPR/Cas13a system can be used to engineer resistances to RNA viruses into crop plants.

## Results

### CRISPR/Cas13a expression in potato plants and target sites selection

**Figure 1:**
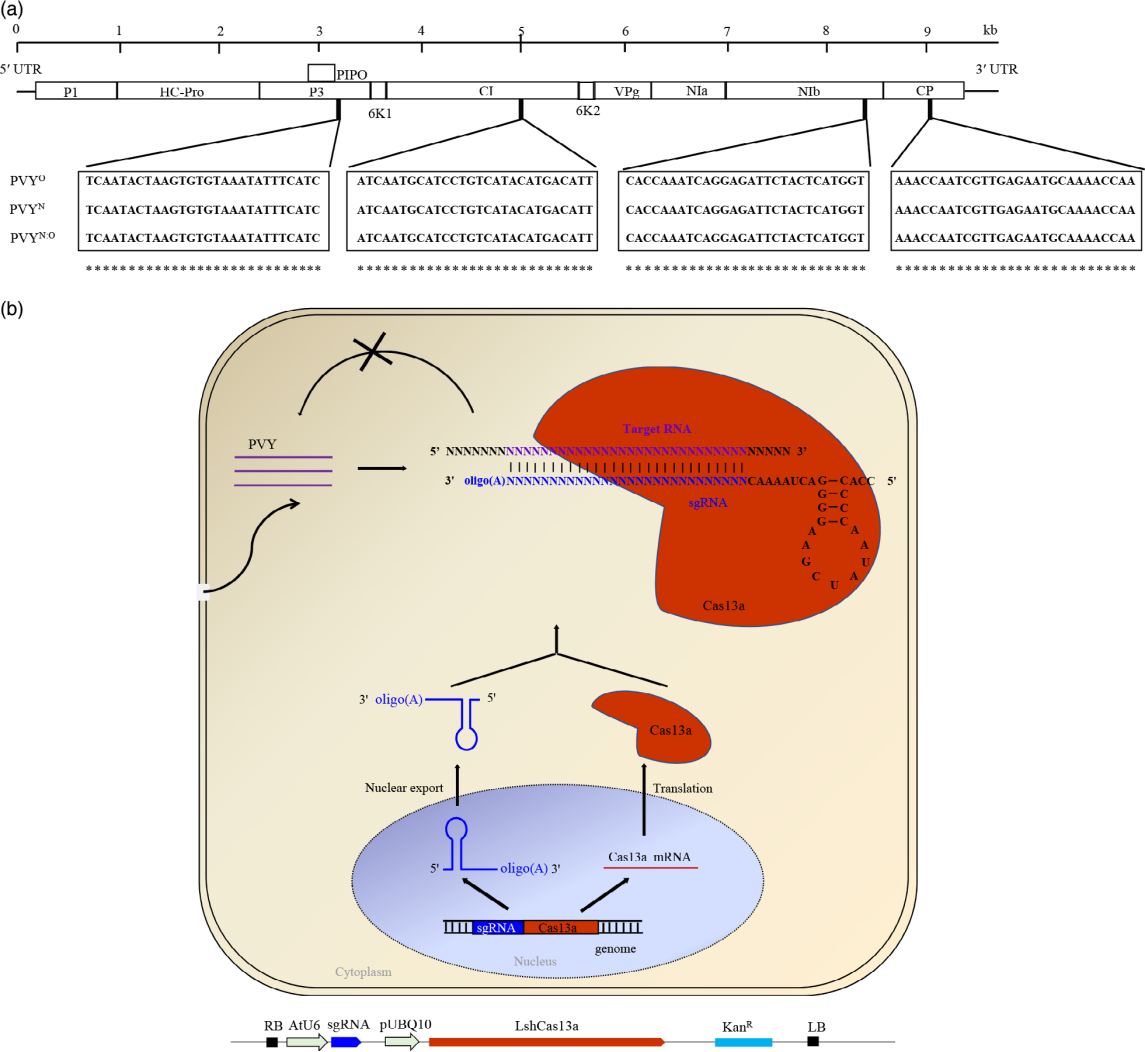
Single guide RNAs (sgRNAs) target sites selection, and overview of the CRISPR/Cas13a for conferring RNA virus resistance in plants. (a) Sequence alignment of three major *Potato virus Y* (PVY) strains: PVY^O^ , PVY^N^ and PVY^N^ ^:O^. sgRNAs were designed based on 100% complementarity to conserved regions in P3, CI, NIb and coat protein among the three PVY strains. (b) Diagrammatic representation of the generation of PVY‐resistant plants with the sequence‐specific sgRNA‐Cas13a system. The expression pathways of the sgRNA and the Cas13a protein and their complex formation to target the RNA genome of PVY are schematically shown. The oligo(A) motif of the sgRNA serves as signal for nuclear export. The physical map of the construct for plant transformation is shown below the model. See text for details.

*Potato virus Y* is prone to high mutation rates and recombination, generating a high level of genetic diversity and a multitude of PVY strains (Davie *et al*., 2017). To target multiple PVY strains, we analysed the genomes of three major PVY strains, PVY^O^, PVY^N^ and the recombinant PVY^N:O^ strain. Fourteen conserved sequences with >28 nucleotide identity (the minimum size of a guide RNA for mRNA targeting by CRISPR/Cas13a) were found. Four of them, targeting the *P3*,* CI*,* NIb* and *CP* regions, respectively, were selected and used for sgRNA design (Figure 1a,b; Table S1). The P3 protein is the potyviral membrane protein involved in virus replication, systemic infection, pathogenicity and movement (Cui *et al*., 2010; Johansen *et al*., 2001; Quenouille *et al*., 2013). The CI protein forms the laminate cytoplasmic inclusion bodies (Edwardson, 1992) involved in virus movement and infection (Carrington *et al*., 1998; Quenouille *et al*., 2013; Wei *et al*., 2010). The NIb protein is the RNA‐dependent RNA polymerase (RdRp) that participates in the replication of the viral RNA (Hong and Hunt, 1996; Quenouille *et al*., 2013). The coat protein (CP) is required for virion assembly, cell‐to‐cell and systemic movement, and aphid transmission (Andersen and Johansen, 1998; Atreya *et al*., 1990; Quenouille *et al*., 2013; Rojas *et al*., 1997).

**Figure 2:**
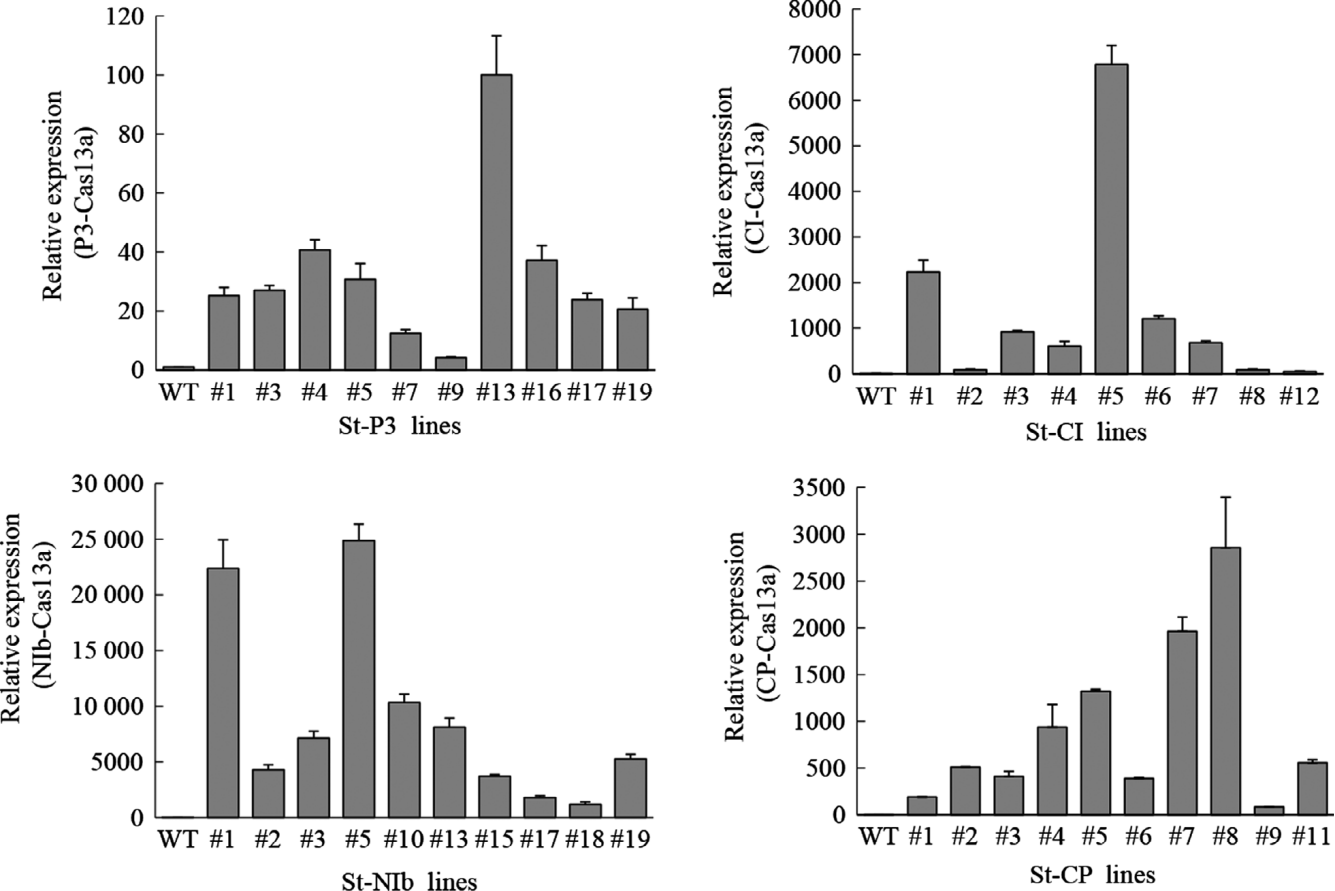
Analysis of the relative expression of the *Cas13a* gene in transgenic lines by quantitative reverse transcriptase‐polymerase chain reaction. The *TUBULIN 2* gene was used as an internal standard. Data are showed as means ± SD (*n* = 3).

Because PVY replicates in the cytoplasm (Kopek *et al*., 2007; Otulak and Garbaczewska, 2013), we constructed binary vectors harbouring a synthetic *LshCas13a* gene driven by the *UBQ10* (*Arabidopsis* ubiquitin‐10) promoter, and *sgRNAs* with oligo (A)‐rich tails (serving as signal for nuclear export) (Stewart, 2010) driven by the *AtU6* promoter. We next introduced the *LshCas13a/sgRNA* constructs into potato plants by stable *Agrobacterium*‐mediated transformation (Figure 1b). Transgenic lines were selected on plant regeneration medium with kanamycin as the selection agent. We obtained over 25 independent transgenic lines for each of the four constructs. Total RNA from ~10 transgenic lines each was extracted, and the expression of *LshCas13a* and the *sgRNAs* was determined by quantitative reverse transcriptase‐polymerase chain reaction (qRT‐PCR). The data revealed that transcript levels of *LshCas13a* correlated well with those of the *sgRNAs* (Figures 2 and S1). In addition, by fusing an HA tag to Cas13a, a positive correlation between mRNA accumulation and protein accumulation of Cas13a could be demonstrated in transient expression assays (Figure S2).

### CRISPR/Cas13a‐mediated interference with PVY

**Figure 3:**
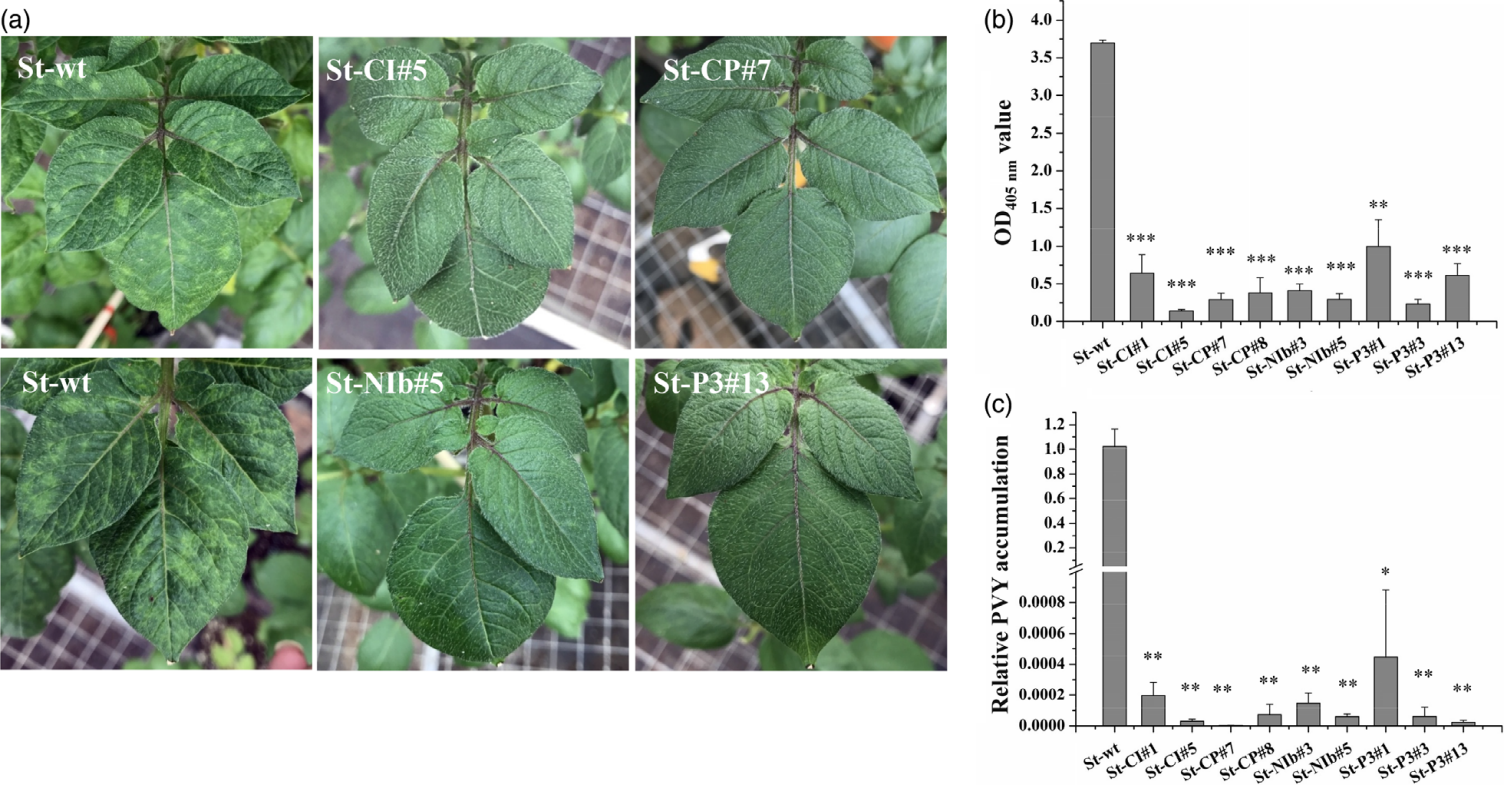
Resistance of transgenic potato plants to PVY^O^ . (a) Symptoms of transgenic and wild‐type *Solanum tuberosum* plants challenged with *Potato virus Y* (PVY) at 25 dpi. Reduction in PVY symptoms is easily visualized on transgenic lines expressing Cas13a/CI‐sgRNA, Cas13a/CP‐sgRNA, Cas13a/NIb‐sgRNA, and Cas13a/CI‐sgRNA. Virus accumulation in transgenic *S. tuberosum* assessed at 25 dpi by enzyme‐linked immunosorbent assays (b) and quantitative reverse transcriptase‐polymerase chain reaction (c). Error bars represent SE. Data represent three biological replicates. Asterisks indicate statistically significant differences (*P < 0.05, **P < 0.01, ***P < 0.001, independent‐samples *t*‐test).

PVY^O^ is a common PVY strain. To test whether our CRISPR/Cas13a constructs interfere with the accumulation of PVY^O^, we selected two transgenic lines with high transgene expression levels for each construct and performed PVY resistance assays. To this end, transgenic and wild‐type (WT) plants were challenged with PVY^O^, and the appearance of disease symptoms was monitored. While the typical PVY mosaic symptoms were observed in leaves of the infected WT plants at 25 days post‐inoculation (dpi), no disease symptoms were observed in any of the transgenic plants (Figure 3a). Resistance of transgenic plants to PVY^O^ was further confirmed by enzyme‐linked immunosorbent assays (ELISA) and qRT‐PCR assays which showed strongly reduced PVY accumulation in systemic leaves of transgenic plants at 20 dpi (Figure S3) and 25 dpi (Figure 3b,c), respectively. These results indicate that transgenic plants accumulate much lower levels of the virus than WT plants after inoculated with PVY.

### The efficiency of PVY inhibition is positively correlated with Cas13a/sgRNA expression levels

**Figure 4:**
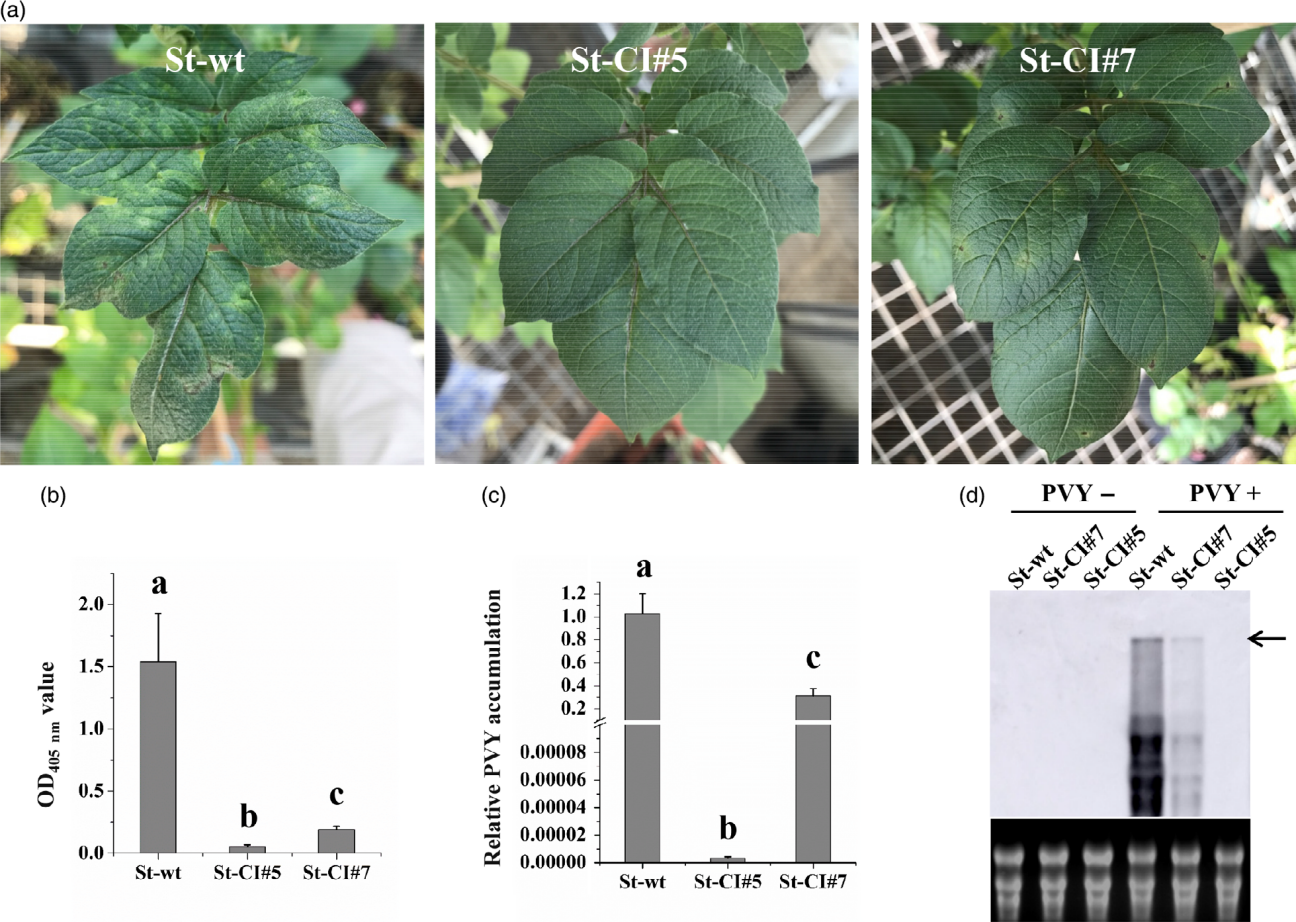
Efficiency of PVY^O^ inhibition correlates with Cas13a/sgRNA expression levels. (a) Disease symptoms in response to PVY^O^ infection in two transgenic *S. tuberosum* lines with high (St‐CI#5) and low (St‐CI#7) levels of *Cas13a/sgRNA* at 25 dpi. Virus accumulation was assessed at 25 dpi by enzyme‐linked immunosorbent assays (b) and quantitative reverse transcriptase‐polymerase chain reaction (c). Error bars represent SE. Data represent three biological replicates. Statistically significant differences are indicated by different letters (*P* < 0.05). (d) Northern blot analysis confirms the correlation between Cas13a/sgRNA expression level and the strength of the interference with PVY^O^ interference. RNA extracted from systemic leaves of infected plants was probed with a DIG‐labelled *Potato virus Y* (PVY) complementary RNA fragment (of 480 nt) targeted to coat protein and detected with an anti‐DIG antibody. The arrow indicates the full‐length PVY genome. The ethidium bromide‐stained rRNAs in the agarose gel prior to blotting is shown below the blot as loading control.

To examine whether the efficiency of PVY^O^ inhibition was correlated with the *LshCas13a/sgRNA* expression levels, one transgenic line with high *LshCas13a/sgRNA* expression level (St‐CI#5; targeting the *CI* region) and one line with relatively low expression level (St‐CI#7) were used for comparison of their resistance levels to PVY^O^ (Figure 2). Twenty‐five days after PVY^O^ infection, severe mosaic symptoms were observed in WT plants. Mild mosaic symptoms were also observed in the transgenic line (St‐CI#7) with low expression level of the *LshCas13a/sgRNA* construct. By contrast, in the transgenic line (St‐CI#5) that expressed high levels of *LshCas13a/sgRNA*, no obvious symptoms were observed (Figure 4a). The correlation between *LshCas13a/sgRNA* expression and inhibited PVY^O^ accumulation was further confirmed by ELISA and qRT‐PCR assays (Figure 4b,c).

To further assess the CRISPR/Cas13a‐mediated interference with the replication of the RNA genome of PVY, total RNA from systemic leaves of PVY‐infected plants was isolated to detect PVY^O^ genome accumulation by northern blotting. Consistent with the results of the ELISA and qRT‐PCR analyses, the data showed that transgenic lines harbouring higher levels of *Cas13a/sgRNA* exhibit stronger interference with viral replication than lines with low level of *LshCas13a/sgRNA* expression (Figure 4d). Together, these data strongly indicate that the efficiency of PVY^O^ inhibition is positively correlated with the *LshCas13a/sgRNA* expression level.

### CRISPR/Cas13a specifically targets PVY genomes

**Figure 5:**
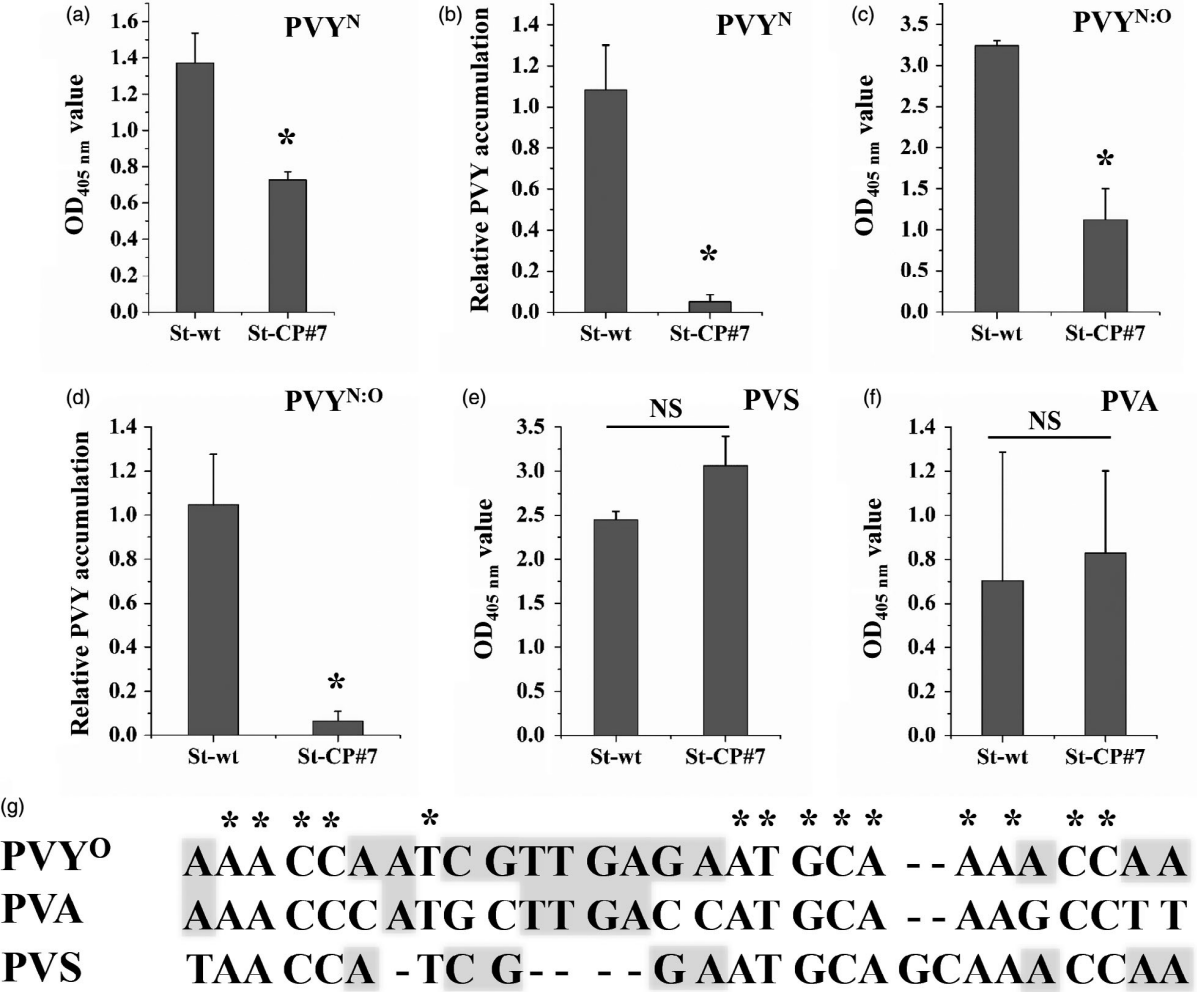
Transgenic potato plants display specific resistance to *Potato virus Y* (PVY). PVY^N^ and PVY^N^ ^:O^ accumulation in transgenic *Solanum tuberosum* plants was assessed 25 dpi by enzyme‐linked immunosorbent assays (ELISA) (a and c) and quantitative reverse transcriptase‐polymerase chain reaction assays (b and d). *Potato virus S* (PVS) (e) and *Potato virus A* (PVA) (f) accumulation in transgenic *S. tuberosum* lines was assessed at 25 dpi by ELISA. Error bars represent SE. Data represent three biological replicates. Asterisks indicate statistically significant differences (*P < 0.05, independent‐samples *t*‐test). (g) Alignment of the CP‐sgRNA‐binding sequence from PVY^O^ with the corresponding regions in the coat protein (CP)‐encoding genes of PVA and PVS. The CP‐sgRNA designed to target PVY shows low sequence similarity to the CP region of PVA and PVS. Shading denotes nucleotides conserved in two viruses, asterisks mark nucleotides conserved in all three viruses.

To test if the CRISPR/Cas13a constructs in our transgenic potato plants also confer resistance to PVY^N^ and the recombinant strain PVY^N:O^ (that both also cause the viral disease), we challenged transgenic plants targeting the CP region of PVY (St‐CP#7) and WT plants with PVY^N^ or PVY^N:O^. Similar to the results obtained upon infection with PVY^O^ (Figure 3), strongly reduced PVY^N^ and PVY^N:O^ accumulation was observed in systemic leaves of the transgenic plants by ELISA and qRT‐PCR assays (Figure 5a–d), indicating that a single sgRNA is capable of targeting multiple PVY strains when appropriately designed (to hit a conserved sequence in the viral genome; Figure 1a).

We next investigated whether our PVY‐resistant transgenic plants also cause interference with other potyviruses such as *Potato virus A* (PVA; He *et al*., 2014) or unrelated viruses such as *Potato virus S* (PVS; Duan *et al*., 2018; Khassanov and Vologin, 2018). After mechanical inoculation with PVS or PVA, transgenic plants and WT plants showed no significant difference in the accumulation of PVS (Figure 5e) or PVA (Figure 5f) viral particles. These results indicate that the sgRNA sequence can be designed with sufficient specificity to target the genome of a single group of viruses (Figures 1a and 5g).

## Discussion

*Potato virus Y* causes one of the most devastating viral diseases in potato plants (Karasev and Gray, 2013; Quenouille *et al*., 2013). Attempts to control the disease with conventional approaches such as breeding and RNA interference have met with limited success (Chaudhary, 2018). Here we demonstrate that the CRISPR/Cas13a immune system can be engineered to generate virus‐resistant potato plants for PVY control.

By generating stable transgenic potato plants, we first aimed to express *LshCas13a/sgRNAs* targeting the *P3*,* CI*,* NIb* or *CP* region of the PVY^O^ genome (Figure 1), and compared their efficiency in interfering with PVY^O^ replication. Our results show that all sgRNAs lead to interference with accumulation of PVY^O^ at similar efficiencies (Figure 3). In transient assays, CRISPR RNAs targeting HC‐Pro and GFP sequences exhibited better interference with Turnip mosaic virus (TuMV) replication than those targeting CP sequence (Aman *et al*., 2018). It remains to be investigated whether this is a specific phenomenon of TuMV and/or the transient sgRNA delivery system used in this study.

By directly comparing the interference efficiency in transgenic lines with high and low expression levels of *LshCas13a/sgRNAs* that target the same *CI* region in the PVY^O^ genome, the efficiency of PVY inhibition was found to be positively correlated with the *LshCas13a/sgRNA* expression level (Figure 4), suggesting that the resistance of engineered plants against PVY^O^ depends on the expression level of the *LshCas13a/sgRNA* construct. Our findings further indicate that a sgRNA designed against a conserved sequence is capable of conferring broad‐spectrum viral resistance by targeting multiple PVY strains. We successfully targeted PVY^O^, PVY^N^ and PVY^N:O^ simultaneously with a sgRNA (Figures 3 and 5a–d), while unrelated viruses (PVA and PVS) that have low sequence similarity with PVY remain unaffected (Figure 5e,f).

*Potato virus Y* replicates in the cytosol. To trigger sgRNA export out of the nucleus, an oligo(A) sequence (of seven adenosines; Data S4) was included immediately adjacent to the spacer sequence. *In vitro* biochemical and crystal structural data have shown that the secondary structure and sequence of the Scaffold region of the sgRNA are critical for interaction with LshCas13a and ssRNA cleavage (Abudayyeh *et al*., 2016; Liu *et al*., 2017a). The Cas13a‐sgRNA complex undergoes a significant conformational change upon target RNA binding. A 20‐bp guide‐target RNA duplex is sufficient to activate Cas13a for efficient cleavage of the target RNA (Liu *et al*., 2017b). A few of unpaired (extra) nucleotides at the 3′ end of the sgRNA do not affect cleavage activity (Abudayyeh *et al*., 2016). Since the extra oligo(A) tail at the 3′ end of sgRNA in our design does not change any of the essential sequence elements of the sgRNA, and is also unlikely disturb the stem‐loop structure of scaffold sequence located at the 5′ end of the sgRNA, targeting of the RNA virus is unlikely to be affected by the oligo(A) sequence. PVY resistance of our transgenic plants supports this conclusion. If presence of the oligo(A) tail would be of any concern, it can be avoided by employing more precise RNA processing tools, such as the Csy4 RNase (Nissim *et al*., 2014), a self‐cleaving ribozyme (Gao and Zhao, 2014; Gao *et al*., 2018; Tang *et al*., 2017) or endogenous tRNA processing enzymes (Xie *et al*., 2015).

Recent work demonstrated the feasibility of simultaneously expressing several sgRNAs by employing the endogenous tRNA‐processing system of plant cells, thus guiding Cas9 to edit multiple chromosomal targets (Xie *et al*., 2015). In nature, plants are exposed to a variety of different viruses. Thus, it would be worthwhile to investigate whether the CRISPR/Cas13a system can be used to express multiple sgRNAs against several RNA viruses to produce multiresistant plants. Multiplexing is also a suitable strategy to prevent (or at least substantially delay) the development of resistance of the virus to cleavage by the CRISPR/Cas13a system. Mutation of the target site of the sgRNA in the viral genome can potentially lead to resistance. This can be counteracted by (i) selecting target sites that have little leeway to mutate and/or (ii) targeting several sites in the genome with two or more sgRNAs.

Besides Cas13a, other RNA‐targeting Cas proteins have been reported. Recently, the CRISPR‐Cas9 system from *Francisella novicida* was reprogrammed to confer molecular immunity against RNA viruses in *N. benthamiana* and *Arabidopsis thaliana* (Zhang *et al*., 2018). The VI‐D Cas13d from *Ruminococcus flavefaciens* was identified as a new RNA‐targeting CRISPR effector (Konermann *et al*., 2018; Yan *et al*., 2018), which is prominently smaller than other Cas13 subtypes and exhibits favourable RNA knock‐down efficiency and specificity compared to RNAi (Konermann *et al*., 2018). All these findings should provide ample opportunities for the development of efficient antiviral strategies based on CRISPR/Cas systems.

Crop plants with virus resistances engineered via the CRISPR/Cas13 system are transgenic plants and, thus, will fall under the current GMO legislation. This means that, similar to insect‐resistant plants expressing Bt toxins, regulatory approval will be required before such varieties can be commercialized. Since potato plants are vegetatively propagated, obtaining regulatory approval may be somewhat less demanding than with sexually propagated plants (that potentially transmit their transgenes via pollen).

In conclusion, we engineered high‐level PVY resistance into potato plants by introducing the CRISPR/Cas13a prokaryotic immune system. Importantly, the CRISPR/Cas13a system, while allowing the specific targeting of viral genomes, can be used to interfere with multiple PVY strains. Together with previously reported transient transformation assays in tobacco (Aman *et al*., 2018), our findings obtained with stably transformed potato plants suggest a new promising strategy to engineer crop plants with resistances to RNA viruses.

## Materials and methods

### Plants, viruses and viral strains

Potato (*S. tuberosum* cultivar Desiree) plants were grown under standard greenhouse conditions as described previously (Zhang *et al*., 2015). The viral strains and isolates used in this study, including PVY^O^‐FL (accession number HM367075, Data S1; Nie *et al*., 2011), PVY^N^‐Jg (accession number AY166867; Nie and Singh, 2003), PVY^N:O^‐Mb112 (accession number AY745491; Nie *et al*., 2004), PVS (accession number KU896946; Wang *et al*., 2016) and PVA (accession number KF977085; He *et al*., 2014) were maintained in tobacco or potato plants as hosts. Strain identity and purity were verified by reverse‐transcription polymerase chain reaction (RT‐PCR) and ELISA as described previously (Wang *et al*., 2016).

### Design and construction of LshCas13a/sgRNA cassettes for the expression in *Solanum tuberosum*

The *Leptotrichia shahii* Cas13a (*LshCas13a*) gene sequence was designed with codon optimization for *S. tuberosum* (Data S2 and S3). The *LshCas13a* gene and the 32 bp sgRNA‐encoding sequence containing two BsaI restriction sites were synthesized by Genecreate (Wuhan, China). The *LshCas13a* gene was linked to the *UBQ10* promoter and the *nos* terminator. The sgRNAs (Data S4) were designed as primer dimers (Table S2) with overhangs, and were cloned under the *Arabidopsis* U6 promoter using the restriction enzymes SacII and SbfI. The final vectors (including *LshCas13a* and the sgRNA expression cassettes) were named pP3, pCI, pNIb and pCP. All cloned sequences were confirmed by resequencing.

### Agro‐infiltration of *Nicotiana benthamiana* leaves

Constructs harbouring the HA‐Cas13a clone were transformed into *Agrobacterium tumefaciens* strain GV3101 by electroporation. Inoculation of leaves with *Agrobacterium* was performed according to previously described protocols (Ji *et al*., 2015). Briefly, overnight‐grown cultures were harvested by centrifugation and suspended in infiltration medium (10 mm MES, 10 mm MgCl_2_, 150 μm acetosyringone). The suspension was diluted to concentrations of OD_600_ 1.0, 0.33, 0.25 and 0.2, and then infiltrated into 4‐week‐old leaves of *N. benthamiana* with a 1 mL needleless syringe.

### Western blot analysis

Cas13a expression was detected by western blot analysis. A commercial anti‐HA antibody (ABclonal, Oxfordshire, UK) was used as primary antibody (1 : 3000 dilution). The signals on the membrane were visualized using the enhanced chemiluminescence substrate (SuperSignal West Pico; Pierce, Rockford, IL) following the manufacturer's instructions.

### Generation of transgenic *Solanum tuberosum* plants

*Agrobacterium tumefaciens* strains harbouring the LshCas13a/sgRNA constructs were employed to transform *S. tuberosum* var. Désirée plants using previously described protocols (Zhang *et al*., 2015). Transgenic plants were identified by their resistance to kanamycin (50 μg/mL) and initially tested for the presence of the transgene by PCR assays. Transgene expression was subsequently confirmed by qRT‐PCR.

### Mechanical inoculation of viruses and ELISA assay

To determine the response of transgenic plants to various viral strains and isolates, wild‐type and transgenic plants were grown in the greenhouse at 20–25 °C. Five to eight plants of each line at the 6–8 leaf stage were infected with inocula of viral strains or isolates (leaf extract: 1 g leaf tissue homogenized in 0.1 M potassium phosphate buffer, pH 7.6) by mechanical wounding as described previously (Nie *et al*., 2004). The same number of wild‐type plants inoculated with the corresponding virus served as control. Symptom development in the potato plants was recorded at 5‐day intervals for 1 month on both the inoculated and the upper uninoculated (systemic) leaves. Double‐antibody sandwich enzyme‐linked immunosorbent assays (DAS‐ELISA) with virus‐specific antibodies (Agdia, Elkhart, IN) were conducted to measure virus accumulation in the upper uninoculated systemic leaves at 15, 20 and 25 days post inoculation (dpi) as described previously (Wang *et al*., 2016).

### RNA extraction and qRT‐PCR analyses

Transcription of *LshCas13a/sgRNA* in transgenic plants was detected by qRT‐PCR assays. Total RNA of potato plants was extracted with the TRIzol reagent (Invitrogen, Carlsbad, CA). cDNA was synthesized using reverse transcriptase and oligo(dT) primer (Takara, Shiga, Japan). qRT‐PCRs were carried out in a CFX96 Touch™ real‐time PCR detection system (Bio‐Rad, Hercules, CA) using SYBR^®^ Premix Ex Taq™ II (Takara). qRT‐PCRs were repeated in three independent experiments. Three technical replicates were performed for each biological replicate. The potato *TUBULIN 2* gene was used as reference. Primer sequences for qRT‐PCR are listed in Table S2.

### Northern blot

Northern blot analyses were performed according to the protocol reported by Zhang *et al*. (2015). A 480 bp RNA probe for PVY was synthesized and labelled using the DIG Northern Starter Kit (Roche, Basel, Switzerland).

### Statistical analyses

The results presented in the figures represent the average of three independent experiments. The significance of the differences between two datasets was assessed by independent‐samples *t*‐test.

## Author's contributions

RB and JZ designed the experiments; XZ, ZZ, RC, YW and BN performed the experiments; XZ, FZ, LC, BN and JZ analysed the results; JZ supervised the project; FZ, RB and JZ wrote the manuscript with input from other authors. All the authors read and approved the final manuscript.

## Conflict of interests

The authors declare that they have no competing interests.

## Supporting information


**Figure S1** Analysis of the relative expression of sgRNAs in transgenic lines by qRT‐PCR.
**Figure S2** Transgenic potato plants display resistance to PVY^O^.
**Figure S3** Determination of Cas13a expression levels in transient assays by qRT‐PCR and western blot analyses.
**Table S1** PVY genome annotation.
**Table S2** List of oligonucleotides used in this study.
**Data S1** PVY^O^ full‐length sequence and sgRNA target sequences.
**Data S2** LshCas13a amino acid sequence.
**Data S3** *LshCas13a* full‐length DNA sequence (codon optimized for expression in the plant nuclear genome).
**Data S4** Sequences of synthetic genes for expression of sgRNAs.
