# Supplementary material for: Generation of virus‐resistant potato plants by RNA genome targeting
Source: Plant Biotechnol J. 2019 Mar 8;17(9):1814–22. doi: 10.1111/pbi.13102 (PMC6686122; doi:10.1111/pbi.13102)
Supplement: Supplementary file 1 — Figure S1 Analysis of the relative expression of sgRNAs in transgenic lines by qRT‐PCR. Figure S2 Transgenic potato plants display resistance to PVYO. Figure S3 Determination of Cas13a expression levels in transient assays by qRT‐PCR and western blot analyses. Table S1 PVY genome annotation. Table S2 List of oligonucleotides used in this study. Data S1 PVYO full‐length sequence and sgRNA target sequences. Data S2 LshCas13a amino acid sequence. Data S3 LshCas13a full‐length DNA sequence (codon optimized for expression in the plant nuclear genome). Data S4 Sequences of synthetic genes for expression of sgRNAs. [file PBI-17-1814-s001.doc]

**Supplemental Information for**

**Generation of virus-resistant potato plants by RNA genome targeting**

Xiaohui Zhan, Fengjuan Zhang, Ziyang Zhong, Ruhao Chen, Yong Wang, Ling Chang, Ralph Bock, Bihua Nie, Jiang Zhang

Corresponding author name*:* Jiang Zhang

**Email:** [zhangjiang@hubu.edu.cn](mailto:zhangjiang@hubu.edu.cn)

**This PDF file includes:**

Figures S1 to S3

Supplementary Sequences 1 to 4

Tables S1 to S2


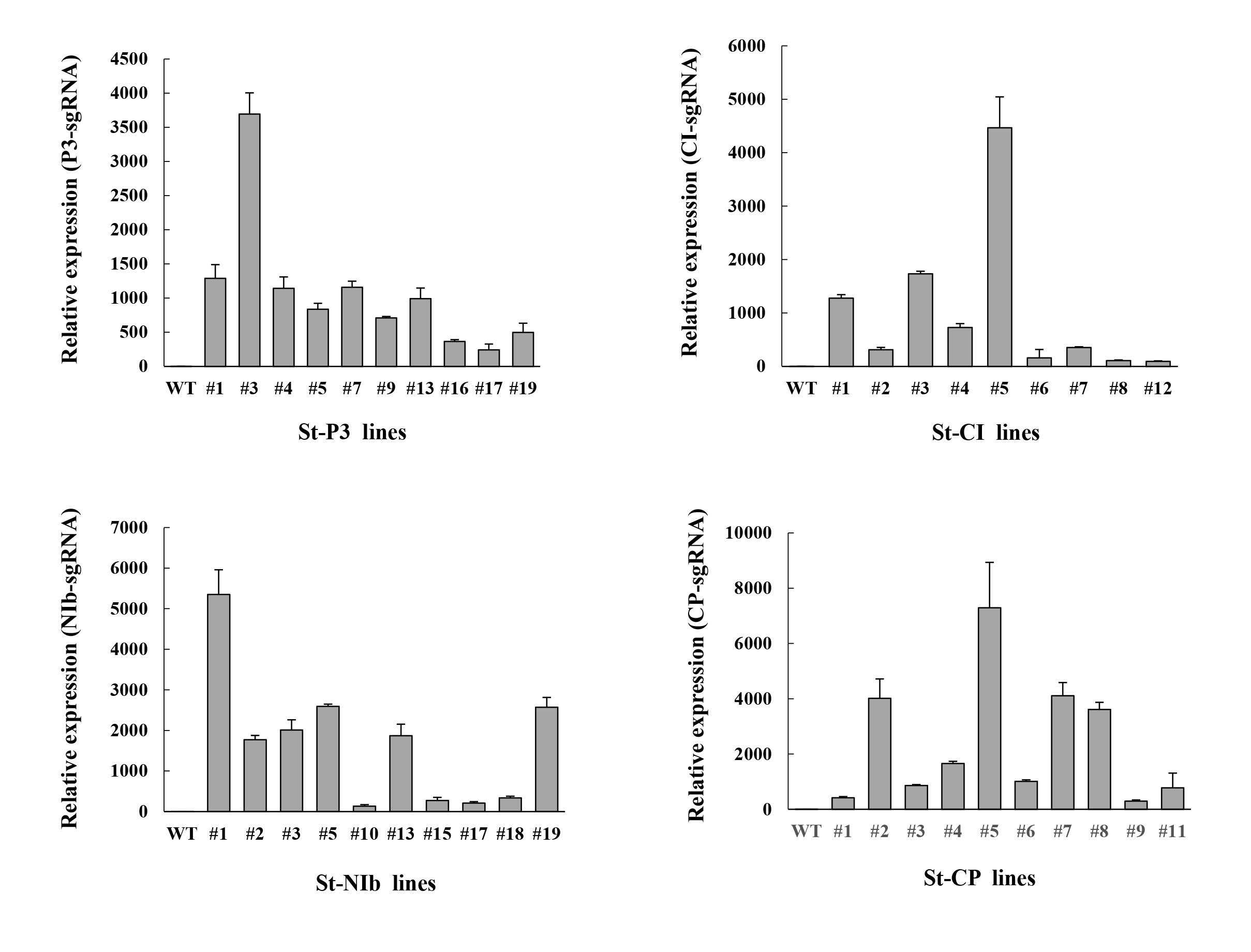


**Figure S1.** Analysis of the relative expression of sgRNAs in transgenic lines by qRT-PCR. The *Tubulin2* gene was used as internal standard. Data are shown as means ± SD (n = 3).


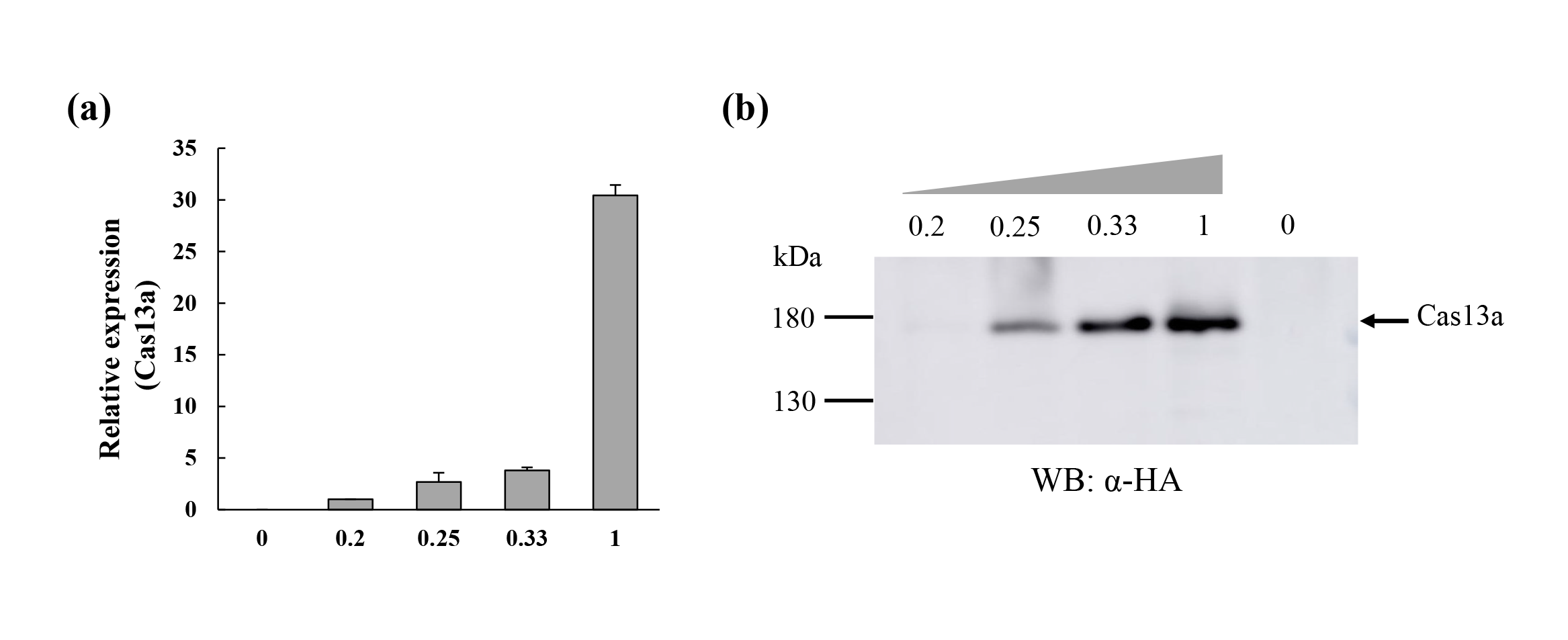


**Figure S2.** **Determination of Cas13a expression levels in transient assays by qRT-PCR (a) and Western blot (b) analyses.** (a) *Cas13a* transcript accumulation as determined by qRT-PCR assays. The numbers on the x-axis (0.2, 0.25, 033, 1.0) indicate the OD_600_ values of diluted *A. tumefaciens* cultures (containing the HA-Cas13a construct) used for infiltration into *N. benthamiana* leaves. 0 indicates the control infiltrated with buffer only. Error bars represent SE (n=3). (b) Immunoblot analysis to determine Cas13a protein accumulation. An anti-HA antibody was used to detect the HA-tagged Cas13a protein


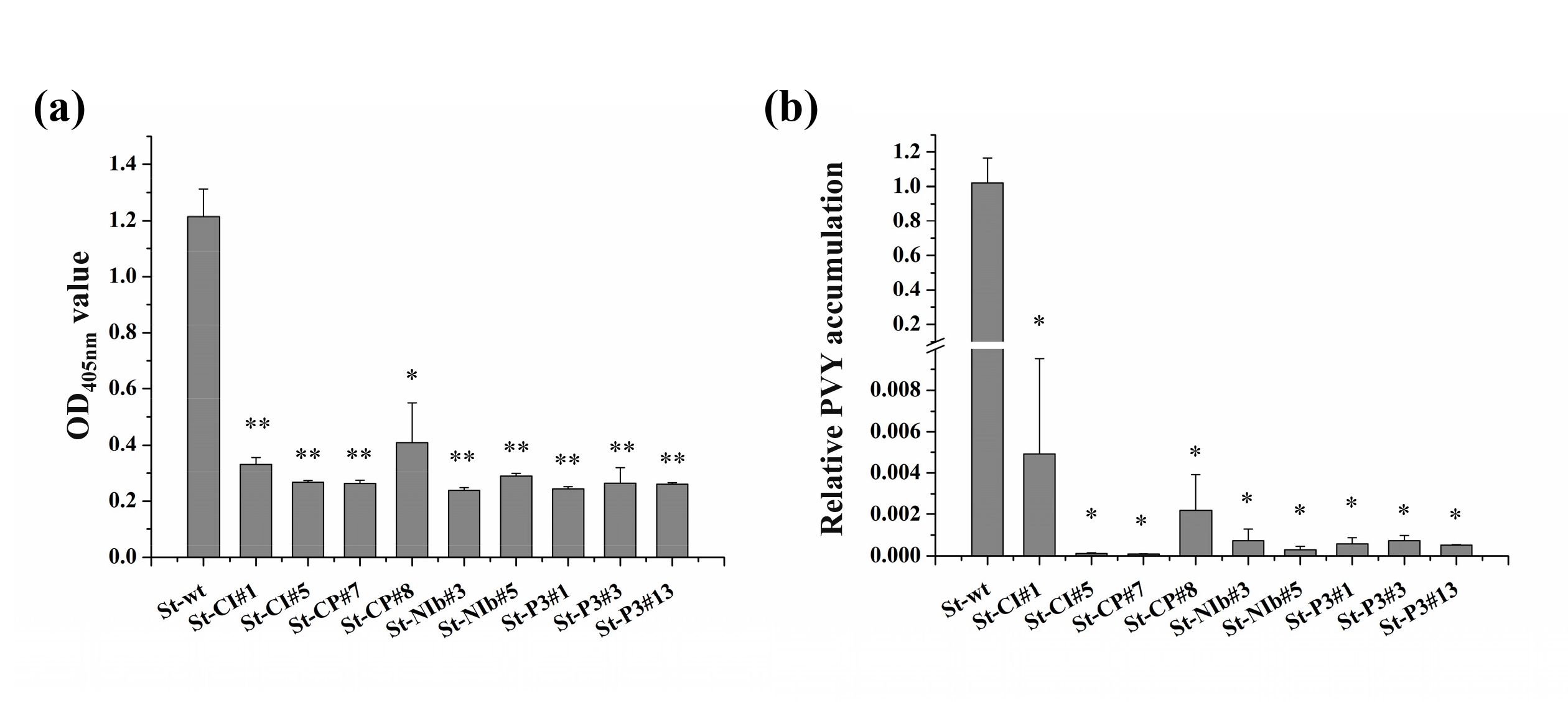


**Figure S3.** Transgenic potato plants display resistance to PVY^O^. Virus accumulation in transgenic *S. tuberosum* plants was assessed 20 dpi by ELISA (a) and qRT-PCR (b). Wild-type plants served as control. Error bars represent SE. Data represent three biological replicates. Asterisks indicate statistically significant differences (*P < 0.05, **P < 0.01, independent-samples *t*-test).

**Sequence S1.** **PVY^O^ full-length sequence and sgRNA target sequences (in different colors).**

AATTAAAACAACTCAATACAACATAAGAAAAACAACGCAAAAACCCTCATAAACGCTTATTCTCACTCAAGCAACTTGTTAAGTTTCAGTTTAAATCATTTCCTTGCAATTCTCTTAAACAATATTGGAAACCGTTTCAACTCAACAAGCAATTTCATCACTTCCGATCAATTTCAGATCCTCAATGGCAACTTACATGTCAACAATCTGTTTCGGTTCGTTTGAATGCAAACTACCATACTCACCCGCCTCTTGCGGGCATATTGTGAAGGAGCGAGAAGTGCTGGCTTCCGTTGATCCTTTCGCAGATCTGGAAACACAACTTAGTGCACGATTGCTCAAGCAAGAATATGCCACTGTTCGTGTGCTCAAGAACGGTACTCTTACTTACCGATACAAGACTGATGCCCAGATAACGCGCATTCAGAAGAAACTGGAGAGGAAGGATAGGGAAGAATATCACTTCCAGATGGCCGCTCCTAGTATTGTGTCAAAAATTACAATAGCTGGTGGAGATCCTCCATCAAAGTCTGAGCCACAAGCACCAAGAGGGATCATTCATACAACTCCAAGGGTGCGTAAAGTCAAGACACGTCCCATAATGAAGTTGACAGAAGGCCAGATGAATCATCTCATTAAGCAGGTGAAGCAGATTATGTCGGAGAAGAGAGGGTCTGTCCACTTAATTAGTAAGAAGACCACTCATGTTCAATATAAGGAGATACTTGGAGCAACTCGCGCAGCGGTTCGAACTGCACATATGATGGGTTTGCGACGGAGAGTGGACTTCCGATGTGATATGTGGACAGTCGGACTTTTGCAACGTCTCGCTCGGACGGACAAATGGTCCAATCAAGTCCGCACTATCAACATACGAAGGGGTGATAGTGGAGTCATTTTGAACACAAAAAGCCTCAAAGGCCACTTTGGTAGAAGTTCAGGAGACTTGTTCATAGTGCGTGGATCACACGAAGGGAAATTGTACGATGCACGTTCTAGAGTTACTCAGAGTGTTTTGAACTCAATGATCCAGTTTTCGAATGCTGATAATTTTTGGAAGGGTCTAGACGGTAATTGGGCACAACTGAGATATCCTTCGGATCACACATGTGTAGCTGGTTTACCTGTCGAAGATTGTGGTAGAGTTGCTGCATTGATGGCACACAGTATCCTCCCGTGCTACAAGATAACCTGCCCCACCTGTGCTCAACAGTATGCCAGCTTGCCGGTTAGCGATCTGTTTAAGCTGTTGCATAAACATGCGAGAGATGGTTTGAACCGATTGGGAGCGGATAAAGACCGGTTTATACATGTTAATAAGTTCTTGATAGCGTTAGAGCATCTAACTGAACCGGTGGATTTGAATCTCGAGCTTTTCAATGAGATATTTAAATCCATAGGGGAGAAGCAGCAAGCACCGTTCAAGAATTTAAATGTCTTAAATAATTTCTTCCTGAAAGGAAAAGAAAATACAGCTCATGAATGGCAAGTGGCTCAATTGAGTTTGCTCGAATTAGCAAGGTTCCAGAAGAATAGAACTGATAATATCAAGAAAGGTGATATATCTTTCTTCAGAAATAAATTATCTGCCAAGGCAAACTGGAATCTGTATTTGTCGTGCGACAACCAGTTGGATAAAAATGCAAATTTTCTGTGGGGACAAAGGGAGTATCATGCTAAGCGGTTTTTCTCAAACTTCTTTGAGGAAATTGATCCAGCAAAGGGATACTCAGCATATGAAATCCGCAAGCATCCAAATGGAACAAGGAAGCTCTCAATTGGTAACTTAGTTGTCCCACTTGATTTAGCTGAGTTTAGGCAGAAGATGAAAGGTGACTATAGGAAACAACCAGGAGTTAGCAGAAAGTGCACGAGTTCGAAAGATGGTAATTATGTGTATCCCTGTTGTTGCACAACACTTGATGATGGTTCAGCTATTGAATCAACATTCTATCCACCAACCAAAAAGCACCTTGTAATAGGCAATAGCGGTGACCAAAAATTTGTTGATTTACCAAAAGGGGATTCGGAGATGTTATACATTGCCAAGCAGGGTTATTGTTATATCAACGTGTTTCTTGCAATGCTTATTAACATTAGCGAGGAGGATGCAAAGGATTTCACAAAGAAAGTTCGCGACATGTGTGTGCCAAAGCTTGGAACCTGGCCAACTATGATGGATTTGGCGACCACTTGTGCTCAAATGAGAATATTCTATCCTGACGTGCATGATGCAGAGCTGCCTAGAATATTGGTTGACCATGACACTCAAACGTGTCACGTGGTTGACTCATTTGGCTCGCAAACAACTGGATATCATATTCTAAAAGCATCCAGCGTGTCTCAACTTATCTTGTTTGCAAATGATGAATTAGAATCTGATATAAAACATTATAGAGTTGGTGGTGTTCCTAATGCATGCCCTGAACTTGGGTCCACAATATCACCTTTCAGAGAAGGAGGAGTTATAATGTCTGAGTCGGCAGCGCTGAAACTGCTTTTAAAGGGAATTTTTAGACCTAAGGTGATGAGACAGTTGCTGTTAGATGAGCCTTACCTGTTGATTTTATCAATATTATCTCCTGGCATACTGATGGCTATGTATAATAATGGGATTTTTGAACTTGCGGTAAGGTTGTGGATTAATGAGAAACAATCCATAGCTATGATAGCATCGCTACTATCAGCTTTAGCCCTACGAGTGTCAGCGGCAGAAACACTCGTCGCACAGAGGATTATCATTGATGCTGCAGCTACAGACCTCCTTGATGCTACGTGTGATGGGTTCAACCTACATCTAACGTACCCCACTGCATTGATGGTGTTGCAAGTTGTTAAGAATAGAAATGAATGTGATGATACCCTATTCAAGGCGGGTTTTTCAAGTTACAACACGAGCGTCGTACAGATTATGGAAAAAAATTATCTAAATCTCTTGAACGATGCTTGGAAAGATTTAACTTGGCGGGAAAAATTATCCGCAACATGGTACTCATACAGAGCAAAACGCTCTATCACTCGGTACATAAAACCCACAGGAAGGGCAGATTTGAAAGGGTTATACAACATATCACCACAAGCATTCTTGGGCCGAAGCGCCCAGGTGGTCAAAGGTACTGCCTCAGGATTGAGTGAGCGATTTAATAATTATT**TCAATACTAAGTGTGTAAATATTTCATC**CTTTTTCATTCGTAGAATCTTTAGGCGTTTGCCAACTTTCGTCACTTTTGTTAACTCATTATTAGTTATTAGTATGTTAACTAGCGTAGTGGCAGTGTGTCAGGCAATAATTTTAGATCAGAGGAAGTATAGGAGAGAAATCGAGTTGATGCAGATAGAGAAGAATGAGATTGTCTGCATGGAGCTATATGCAAGTTTACAGCGCAAACTTGAACGCGATTTCACATGGGATGAGTACATTGAGTATTTGAAATCAGTAAACCCTCAGATAGTTCAGTTTGCTCAAGCGCAGATGGAAGAATATGATGTGCGACACCAGCGTTCCACACCAGGTGTTAAAAATTTGGAACAAGTGGTAGCATTTATGGCTTTAGTCATTATGGTGTTCGATGCTGAAAGGAGTGATTGCGTGTTCAAAACTCTCAATAAATTTAAGGGTGTCCTTTCCTCAATGGACTATGAAGTTAGACATCAGTCCTTAGACGATGTGATCAAGAATTTTGATGAGAGGAATGAGATTATTGATTTTGAATTGAGTGAGGACACAATTCGAACATCATCAGTGCTAGATACAAAGTTTAGTGATTGGTGGGACCGACAAATCCAGATGGGACATACACTTCCACATTACAGAACCGAGGGGCACTTCATGGAATTCACAAGAGCAACTGCTGTCCAAGTGGCTAATGACATTGCCCATAGCGAACACCTAGACTTTCTAGTAAGGGGAGCTGTTGGGTCTGGAAAGTCAACTGGGTTACCTGTTCATCTTAGTGTAGCCGGATCTGTGCTTTTAATTGAACCAACGCGACCACTAGCGGAGAACGTTTTCAAACAGCTATCTAGTGAACCATTCTTCAAGAAGCCAACACTGCGTATGCGTGGAAATAGTATATTTGGCTCTTCTCCAATCTCCGTCATGACTAGCGGATTTGCGCTACACTACTTCGCCAATAATCGCTCTCAATTAGCTCAGTTCAACTTTGTAATATTTGATGAGTGCCATGTTCTGGATCCCTCCGCAATGGCGTTCCGCAGTCTGCTGAGTGTTTATCATCAAGCATGCAAAGTATTAAAAGTGTCAGCTACTCCAGTGGGAAGAGAGGTTGAATTTACAACACAGCAACCAGTCAAGTTAATAGTGGAGGACACACTGTCTTTCCAATCATTTGTTGATGCACAAGGTTCTAAAACTAATGCTGATGTTGTTCAGTTTGGTTCAAACGTACTTGTGTACGTGTCGAGCTACAATGAAGTTGATACCTTGGCTAAGCTCCTAACAGATAAGAATATGATGGTCACAAAGGTTGATGGCAGAACAATGAAGCACGGTTGCCTAGAAATTGTCACAAAAGGAACCAGTGCGAGACCACATTTTGTTGTAGCAACCAACATAATTGAGAATGGAGTGACTTTGGACATAGACGTGGTTGTAGATTTTGGGTTAAAAGTCTCACCGTTCTTGGACATTGACAATAGGAGCATTGCTTACAATAAGGTGAGTGTTAGCTATGGTGAGAGAATTCAAAGGCTGGGTCGTGTTGGACGCTTCAAGAAAGGAGTAGCATTGCGCATTGGACACACTGAGAAGGGAATTATTGAAATTCCAAGCATGATCGCTACAGAGGCAGCTCTTGCTTGCTTTGCATATAACTTACCAGTGATGACAGGAGGCGTCTCAACTAGTCTGATTGGCAATTGTACTGTGCGCCAAGTTAAAACAATGCAGCAATTTGAATTGAGTCCCTTCTTTATCCAGAATTTCGTTGCCCATGATGG**ATCAATGCATCCTGTCATACATGACATT**CTTAAAAAGTATAAACTTCGAGATTGTATGACACCTTTGTGCGATCAGTCTATACCATACAGGGCATCGAGCACTTGGTTATCGGTTAGTGAATATGAGCGACTTGGAGTGGCCTTAGAAATTCCAAAGCAAGCCAAAATTGCATTCCATATCAAAGAGATCCCTCCTAAGCTCCACGAAATGCTTTGGGAAACGGTTGTCAAGTACAAAGACGTTTGCTTATTTCCAAGCATTCGAGCATCGTCCATCAGCAAAATCGCATACACATTGCGTACAGACCTCTTCGCCATCCCAAGAACTCTAATATTGGTGGAGAGACTGCTTGAAGAGGAGCGAGTGAAGCAGAGCCAATTCAGAAGTCTCATCGATGAAGGATGCTCAAGCATGTTTTCAATTGTCAACCTGACAAACACTCTCAGAGCTAGATATGCAAAAGATTACACCGCAGAGAACATACAAAAACTTGAGAAAGTGAGAAGTCAATTGAAAGAATTCTCAAATTTGGATGGTTCTGCATGTGAGGAAAATTTAATAAAGAGGTATGAGTCTTTGCAGTTCGTTCATCACCAAGCTGCGACGTCACTTGCAAAGGATCTCAAGTTGAAGGGGACTTGGAAGAAGTCATTGGTGGCCAAAGACTTGATCATAGCAGGCGCTGTTGCAATTGGTGGAATAGGACTCATATATAGTTGGTTCACACAATCAGTTGAGACTGTGTCTCACCAAGGGAAAAATAAATCCAAAAGAATTCAAGCCTTGAAGTTTCGCCATGCTCGTGACAAAAGGGCTGGCTTTGAAATTGACAACAATGATGACACAATAGAGGAATTCTTTGGATCTGCATACAGGAAAAAGGGAAAAGGTAAAGGTACCACAGTTGGTATGGGCAAGTCAAGCAGGAGGTTCATCAACATGTATGGGTTTGATCCAACAGAGTACTCATTCATCCAATTCGTTGATCCACTCACTGGGGCGCAAATAGAAGAGAATGTCTATGCTGACATTAGAGATATTCAAGAGAGATTTAGTGAAGTGCGAAAGAAAATGGTTGAGAATGATGACATTGAAATGCAAGCCTTGGGTAGTAACACGACCATACATGCATACTTTAGAAAAGATTGGTCTGACAAAGCTTTGAAGATTGATTTAATGCCACATAATCCACTCAAAGTTTGTGACAAGACAAATGGCATTGCCAAATTTCCTGAGAGAGAGCTCGAACTAAGGCAGACTGGGCCAGCTGTAGAAGTCGATGTGAAGGACATACCAGCACAGGAGGTGGAGCATGAAGCTAAATCGCTCATGAGAGGCTTGAGAGACTTCAATCCAATTGCCCAAACAGTTTGTAGGCTGAAAGTATCTGTTGAATATGGGACATCAGAGATGTACGGTTTTGGATTTGGAGCATACATAATAGCGAACCACCATTTATTCAGGAGTTACAATGGTTCCATGGAGGTGCGATCCATGCACGGTACATTCAGGGTGAAGAATCTACACAGTTTGAGCGTTCTGCCAATTAAAGGTAGGGATATCATCCTCATCAAAATGCCGAAAGATTTCCCTGTCTTTCCACAGAAATTGCATTTCCGAGCTCCTACACAGAATGAAAGAGTTTGTTTAGTTGGAACCAACTTTCAGGAGAAGTATGCATCGTCGATCATCACAGAAACAAGCACCACTTACAATATACCAGGCAGCACATTCTGGAAGCATTGGATTGAAACAGATAATGGACATTGTGGATTACCAGTGGTGAGCACCACCGATGGATGTCTAGTCGGAATTCACAGTTTGGCAAACAACAAACACACCACGAACTACTACTCAGCCTTTGATGAAGATTTTGAAAGCAAGTATCTCCGAACCAATGAGCACAATGAATGGGTCAAGTCTTGGATTTATAATCCAGACACAGTGTTGTGGGGCCCGTTGAAACTTAAAGACAGCACTCCCAAAGGATTATTCAAAACAACAAAGCTTGTGCAAGATCTAATCGATCATGATGTAGTGGTGGAGCAAGCTAAGCACTCTGCGTGGATGTTTGAAGCTTTGACAGGAAATTTGCAAGCTGTCGCAACAATGAAGAGCCAATTAGTAACCAAGCATGTAGTTAAAGGAGAGTGTCGACACTTCAAAGAATTCCTGACTGTGGATGCAGAAGCAGAGGCATTCTTCAGGCCTTTGATGGATGCGTATGGGAAAAGCTTGCTGAATAGAGATGCATACATCAAGGACATAATGAAGTATTCAAAACCTATAGATGTTGGTATCGTGGACTGTGATGCATTTGAGGAAGCCATCAATAGGGTTATTATCTACCTGCAAGTGCACGGCTTCAAGAAGTGCGCATACGTCACTGACGAGCAAGAAATTTTCAAAGCGCTCAACATGAAAGCTGCAGTTGGAGCCATGTATGGTGGCAAAAAGAAAGACTATTTTGAGCATTTCACTGATGCAGATAAGGAAGAAATAGTCATGCAAAGCTGTCTGCGATTGTATAAAGGCTTGCTTGGCATTTGGAATGGATCATTGAAGGCAGAGCTCCGGTGTAAGGAAAAGATACTTGCAAATAAGACGAGGACATTCACTGCTGCACCTTTAGACACTTTGCTGGGTGGTAAAGTGTGTGTTGATGATTTCAATAATCAATTTTATTCAAAGAATATTGAATGCTGTTGGACGGTTGGGATGACTAAGTTTTATGGTGGTTGGGATAAACTGCTGCGGCGTTTACCTGAGAATTGGGTATACTGTGATGCCGATGGCTCACAGTTTGATAGTTCACTAACTCCATACTTAATCAATGCTGTTCTCACCATCAGAAGCACATACATGGAAGATTGGGATGTGGGGTTGCAAATGTTGCGCAATTTATACACTGAGATTGTTTACACACCTATTTCAACTCCAGATGGAACAATTGTTAAGAAGTTCAGAGGAAATAACAGTGGTCAGCCTTCTACTGTTGTGGACAACTCTCTTATGGTCGTCCTTGCCATGCACTATGCTCTCATCAAAGAATGCATTGAGTTTGAAGAGATTGACAGCACGTGCGTGTTCTTTGTCAATGGTGATGATTTGCTGATCGCTGTGAATCCGGATAAAGAGGGCATTCTTGACAGATTGTCACAACACTTCTCAGATCTTGGTTTGAATTATGATTTCTCGTCAAGAACAAGAAATAAGGAGGAATTGTGGTTTATGTCTCATAGAGGTCTACTGATTGAGGGCATGTACGTGCCGAAACTTGAAGAAGAAAGGATTGTGTCCATTCTCCAATGGGACAGAGCAGACTTGGCTGAACACAGGCTTGAGGCGATTTGCGCAGCTATGATAGAGTCCTGGGGTTATTCTGAACTAACA**CACCAAATCAGGAGATTCTACTCATGGT**TATTGCAACAGCAACCCTTTGCAACAATAGCGCAGGAAGGGAAGGCTCCTTATATAGCAAGCATGGCATTAAGGAAATTGTATATGGATAGGGCTGTGGATGAGGAAGAGCTGAGAGCCTTCACTGAAATGATGGTCGCATTAGACGATGAGTTTGAATTTGACTCTTATGAAGTACACCATCAAGCAAATGACACAATCGATGCAGGAGGAAGCAGCAAGAAAGATGCAAGACCGGAGCAAGGCAGCATCCAGTCAAACCCGAACAAAGGAAAAGATAAGGATGTGAATGCTGGTACATCTGGGACACATACTGTGCCGAGAATCAAGGCTATCACGTCCAAAATGAGAATGCCCAAAAGCAAGGGAGCAACCGTGCTAAACCTAGAACACTTGCTTGAGTATGCTCCACAACAAATTGATATTTCAAATACTCGGGCAACTCAATCACAGTTTGATACGTGGTATGAGGCAGTGCGGATGGCATACGACATAGGAGAAACTGAGATGCCAACTGTGATGAATGGGCTTATGGTTTGGTGCATTGAAAATGGAACCTCGCCAAATGTCAACGGAGTTTGGGTTATGATGGATGGGAATGAACAAGTCGAGTACCCGTTG**AAACCAATCGTTGAGAATGCAAAACCAA**CCCTTAGGCAAATCATGGCACATTTCTCAGATGTTGCAGAAGCGTATATAGAAATGCGCAACAAAAAGGAACCATATATGCCACGATATGGTTTAATTCGAAATCTGCGGGATGTGGGTTTAGCGCGTTATGCCTTTGACTTTTATGAGGTCACATCACGAACACCAGTGAGGGCTAGGGAAGCGCACATTCAAATGAAGGCCGCAGCATTGAAATCAGCCCAACCTCGACTTTTCGGGTTGGACGGTGGCATCAGTACACAAGAGGAGAACACAGAGAGGCACACCACCGAGGATGTCTCTCCAAGTATGCATACTCTACTTGGAGTCAAGAACATGTGATGTAGTGTCTCTCCGGACGATATATAAGTATTTACATATGCAGTAAGTATTTTGGCTTTTCCTGTACTACTTTTATCATAATTAATAATCAGTTTGAATATTACTAATAGATAGAGGTGGCAGGGTGATTTCGTCATTGTGGTGACTCTATCTTTTAATTCCGCATTATTAAGTCTTAGATAAAAGTGCCGGGTTGTCGTTGTTGTGGATGATTCATCGATTAGGTGATGTCGCGATTTTGTCGTAGCAGTGACTATGTCTGGATCTATCTGCTTGGGTGGTGTTGTGATTTCGTCATAACAGTGACTGTAAACTTCAATCAGGAGAC

The sequences are annotated as follows:

Red nucleotides: P3-sgRNA target sequence; Blue nucleotides: CI-sgRNA target sequence; Green nucleotides: NIb-sgRNA target sequence; Purple nucleotides: CP-sgRNA target sequence.

**Sequence S2. LshCas13a amino acid sequence.**

MGNLFGHKRWYEVRDKKDFKIKRKVKVKRNYDGNKYILNINENNNKEKIDNNKFIRKYINYKKNDNILKEFTRKFHAGNILFKLKGKEGIIRIENNDDFLETEEVVLYIEAYGKSEKLKALGITKKKIIDEAIRQGITKDDKKIEIKRQENEEEIEIDIRDEYTNKTLNDCSIILRIIENDELETKKSIYEIFKNINMSLYKIIEKIIENETEKVFENRYYEEHLREKLLKDDKIDVILTNFMEIREKIKSNLEILGFVKFYLNVGGDKKKSKNKKMLVEKILNINVDLTVEDIADFVIKELEFWNITKRIEKVKKVNNEFLEKRRNRTYIKSYVLLDKHEKFKIERENKKDKIVKFFVENIKNNSIKEKIEKILAEFKIDELIKKLEKELKKGNCDTEIFGIFKKHYKVNFDSKKFSKKSDEEKELYKIIYRYLKGRIEKILVNEQKVRLKKMEKIEIEKILNESILSEKILKRVKQYTLEHIMYLGKLRHNDIDMTTVNTDDFSRLHAKEELDLELITFFASTNMELNKIFSRENINNDENIDFFGGDREKNYVLDKKILNSKIKIIRDLDFIDNKNNITNNFIRKFTKIGTNERNRILHAISKERDLQGTQDDYNKVINIIQNLKISDEEVSKALNLDVVFKDKKNIITKINDIKISEENNNDIKYLPSFSKVLPEILNLYRNNPKNEPFDTIETEKIVLNALIYVNKELYKKLILEDDLEENESKNIFLQELKKTLGNIDEIDENIIENYYKNAQISASKGNNKAIKKYQKKVIECYIGYLRKNYEELFDFSDFKMNIQEIKKQIKDINDNKTYERITVKTSDKTIVINDDFEYIISIFALLNSNAVINKIRNRFFATSVWLNTSEYQNIIDILDEIMQLNTLRNECITENWNLNLEEFIQKMKEIEKDFDDFKIQTKKEIFNNYYEDIKNNILTEFKDDINGCDVLEKKLEKIVIFDDETKFEIDKKSNILQDEQRKLSNINKKDLKKKVDQYIKDKDQEIKSKILCRIIFNSDFLKKYKKEIDNLIEDMESENENKFQEIYYPKERKNELYIYKKNLFLNIGNPNFDKIYGLISNDIKMADAKFLFNIDGKNIRKNKISEIDAILKNLNDKLNGYSKEYKEKYIKKLKENDDFFAKNIQNKNYKSFEKDYNRVSEYKKIRDLVEFNYLNKIESYLIDINWKLAIQMARFERDMHYIVNGLRELGIIKLSGYNTGISRAYPKRNGSDGFYTTTAYYKFFDEESYKKFEKICYGFGIDLSENSEINKPENESIRNYISHFYIVRNPFADYSIAEQIDRVSNLLSYSTRYNNSTYASVFEVFKKDVNLDYDELKKKFKLIGNNDILERLMKPKKVSVLELESYNSDYIKNLIIELLTKIENTNDTL*

**Sequence S3. *LshCas13a* full-length DNA sequence (codon optimized for expression in the plant nuclear genome).**

ATGGGAAACCTCTTCGGACACAAGAGATGGTACGAGGTGAGGGACAAGAAGGACTTCAAGATCAAGCGTAAGGTGAAGGTGAAGAGGAACTACGACGGGAACAAGTACATCCTCAACATCAACGAGAACAACAACAAAGAGAAGATCGATAACAACAAGTTCATCCGTAAGTACATCAACTACAAGAAGAACGACAACATCCTGAAAGAGTTCACGAGGAAGTTCCACGCCGGAAACATCCTCTTCAAGCTCAAGGGAAAAGAGGGGATCATCAGGATTGAGAACAACGACGACTTCCTCGAGACTGAAGAGGTGGTGCTTTACATCGAGGCTTACGGGAAGTCTGAGAAGCTTAAGGCTCTCGGGATCACCAAGAAGAAGATCATCGACGAGGCTATCAGGCAGGGAATCACCAAGGATGACAAGAAGATTGAGATCAAGAGGCAAGAGAACGAGGAAGAGATCGAGATCGATATCAGGGACGAGTACACCAACAAGACCCTCAACGACTGCTCTATCATCCTCCGTATCATCGAGAACGACGAGCTTGAGACTAAGAAGTCCATCTACGAGATCTTCAAGAACATCAACATGAGCCTCTACAAGATTATCGAGAAAATTATCGAAAACGAGACTGAGAAGGTGTTCGAGAACAGGTACTACGAGGAACACCTCCGTGAGAAGCTCCTCAAGGACGATAAGATCGATGTGATCCTCACCAACTTCATGGAAATTCGTGAGAAGATCAAGTCCAACCTCGAGATCCTCGGCTTCGTGAAGTTCTACCTTAACGTTGGCGGGGATAAGAAGAAGTCGAAGAACAAGAAAATGCTGGTCGAGAAGATCTTGAACATTAACGTGGACCTCACCGTCGAGGATATCGCTGACTTCGTGATCAAAGAGCTTGAGTTCTGGAACATCACCAAGCGTATCGAGAAGGTGAAAAAGGTGAACAACGAGTTCCTTGAGAAGAGGCGTAACAGGACCTATATCAAGAGCTACGTGCTCCTCGACAAGCACGAGAAGTTTAAGATCGAGCGTGAGAACAAAAAGGACAAGATCGTCAAGTTCTTCGTGGAAAACATCAAGAACAACTCCATCAAAGAGAAAATTGAGAAGATTCTCGCCGAGTTCAAGATTGACGAGCTGATCAAGAAGCTCGAGAAAGAGCTGAAGAAGGGCAACTGCGACACTGAGATCTTCGGGATTTTCAAGAAGCACTACAAGGTCAACTTCGACAGCAAGAAGTTCTCCAAGAAGTCCGACGAAGAGAAAGAGTTGTACAAGATCATCTACCGTTACCTCAAGGGGCGTATCGAAAAGATCCTTGTGAACGAGCAGAAGGTGAGGCTCAAGAAGATGGAAAAGATTGAAATAGAGAAAATCCTCAACGAGAGCATCCTCTCGGAGAAGATCCTTAAGAGGGTGAAGCAGTACACCCTCGAGCACATCATGTACCTCGGAAAGCTCAGGCACAACGACATCGATATGACTACCGTGAACACGGACGACTTCTCTAGGCTCCATGCCAAAGAAGAGTTGGACCTCGAGCTTATCACCTTCTTCGCCAGCACTAACATGGAACTCAACAAGATATTCAGCCGAGAGAATATCAACAACGATGAGAACATCGACTTTTTCGGCGGCGACCGAGAGAAGAACTACGTTCTCGACAAAAAGATTCTCAACTCTAAGATCAAGATCATTAGGGACCTCGACTTCATCGACAACAAGAACAATATCACCAACAACTTCATCAGAAAGTTCACCAAGATCGGGACCAACGAGAGGAACAGAATCCTCCACGCTATCAGCAAAGAGAGGGATCTCCAGGGAACCCAGGACGACTATAACAAGGTGATCAACATCATCCAGAACCTGAAGATCAGTGACGAAGAGGTGAGCAAGGCTCTCAACCTCGATGTGGTGTTCAAGGATAAGAAAAACATCATCACGAAGATTAACGACATCAAGATCTCCGAGGAAAACAACAACGATATCAAGTACCTGCCGAGCTTCAGCAAGGTTCTCCCTGAGATTCTTAACCTCTACCGTAACAACCCGAAGAACGAGCCTTTCGACACTATCGAGACAGAAAAGATCGTGCTCAACGCCCTCATCTACGTGAACAAAGAACTCTACAAAAAGCTCATCTTGGAGGACGACCTTGAGGAAAACGAGTCCAAGAACATATTCCTGCAAGAGCTTAAGAAAACCCTCGGGAACATCGATGAGATCGACGAGAACATCATTGAGAACTATTACAAGAACGCCCAGATCAGCGCCAGCAAGGGAAACAACAAGGCCATCAAGAAGTACCAGAAAAAGGTCATCGAGTGCTACATCGGCTACCTCCGTAAGAACTATGAGGAACTGTTCGACTTCAGCGATTTCAAGATGAACATCCAAGAGATCAAAAAGCAGATCAAGGATATCAATGATAACAAGACCTACGAGCGTATTACCGTCAAGACCAGCGATAAGACCATCGTCATCAACGATGACTTCGAGTACATTATCTCTATCTTCGCCCTCCTCAACTCCAACGCGGTGATTAACAAGATCCGTAACCGTTTCTTCGCGACCTCTGTGTGGCTTAACACTAGCGAGTACCAAAACATTATCGACATCCTGGACGAGATCATGCAGCTCAACACTCTCAGAAACGAGTGCATCACCGAGAACTGGAACCTGAACCTCGAAGAGTTCATCCAAAAGATGAAGGAAATCGAAAAGGACTTTGACGACTTTAAGATCCAGACCAAGAAAGAGATTTTCAACAACTATTACGAGGATATTAAGAACAACATCTTGACTGAGTTTAAGGACGACATTAACGGGTGCGACGTGCTCGAAAAGAAGTTGGAGAAAATCGTCATCTTCGACGACGAGACTAAGTTCGAAATCGACAAGAAGTCTAACATCCTCCAGGACGAGCAGAGGAAGCTCAGCAACATTAACAAAAAAGACCTTAAAAAGAAGGTGGACCAGTACATTAAGGACAAGGACCAAGAGATTAAGAGCAAGATCCTCTGCAGGATCATCTTCAACAGCGACTTTCTCAAAAAGTATAAGAAAGAAATTGACAACCTCATCGAGGACATGGAAAGCGAGAATGAGAACAAGTTTCAAGAGATCTACTACCCGAAAGAGCGTAAGAACGAACTGTACATCTACAAGAAAAACCTGTTCCTGAACATCGGGAACCCGAATTTCGACAAGATCTACGGGCTCATCAGCAATGACATTAAGATGGCCGACGCCAAGTTCCTGTTCAACATCGACGGAAAGAACATTAGGAAGAACAAAATCAGCGAGATTGACGCCATCCTGAAGAACCTTAACGACAAGCTCAACGGGTACTCCAAAGAGTACAAAGAAAAGTATATCAAAAAGTTGAAAGAGAATGACGACTTCTTTGCCAAGAACATCCAGAACAAGAACTACAAGAGCTTCGAGAAGGACTACAACCGTGTGAGCGAGTACAAGAAGATCAGGGACCTCGTCGAGTTCAACTACCTGAACAAGATCGAGAGCTACCTGATCGACATCAACTGGAAGCTCGCTATCCAGATGGCTAGGTTCGAGAGGGATATGCACTACATCGTGAACGGACTTCGAGAGCTGGGCATCATCAAGCTCTCAGGATACAACACCGGAATCAGCAGGGCTTACCCTAAGAGGAATGGAAGCGACGGATTCTACACTACCACCGCCTACTACAAGTTTTTCGACGAGGAAAGCTATAAGAAGTTTGAAAAGATCTGTTACGGCTTCGGGATCGACCTCTCTGAGAACAGTGAAATCAACAAGCCTGAGAACGAGTCTATCCGAAACTACATCAGCCACTTCTACATCGTTAGGAACCCGTTCGCCGACTACTCTATCGCTGAGCAGATCGATAGAGTGAGCAACCTGCTCTCTTACAGCACCAGGTACAACAACTCGACCTACGCCTCTGTTTTCGAAGTGTTCAAGAAGGATGTTAACCTCGACTACGACGAGTTGAAGAAGAAATTCAAGCTGATCGGGAACAATGACATACTCGAGCGACTCATGAAGCCTAAGAAGGTGTCAGTTCTCGAGTTGGAGAGCTACAACTCCGACTACATCAAAAACCTCATCATCGAGCTGCTCACCAAAATCGAGAACACTAACGATACCCTGTGA

**Sequence S4. Sequences of synthetic genes for expression of sgRNAs.**

P3-sgRNA:

**G**CCACCCCAATATCGAAGGGGACTAAAACGATGAAATATTTACACACTTAGTATTGA*AAAAAAA*TTTTTT

CI-sgRNA:

**G**CCACCCCAATATCGAAGGGGACTAAAACAATGTCATGTATGACAGGATGCATTGAT*AAAAAAA*TTTTTT

Nib-sgRNA:

**G**CCACCCCAATATCGAAGGGGACTAAAACACCATGAGTAGAATCTCCTGATTTGGTG*AAAAAAA*TTTTTT

CP-sgRNA:

**G**CCACCCCAATATCGAAGGGGACTAAAACTTGGTTTTGCATTCTCAACGATTGGTTT*AAAAAAA*TTTTTT

The sequences are annotated as follows:

**G** (bold): transcription initiation site; Yellow background: scaffold sequence; Red nucleotides: P3-sgRNA spacer; Blue nucleotides: CI-sgRNA spacer; Green nucleotides: NIb-sgRNA spacer; Purple nucleotides: CP-sgRNA spacer; Letters in italic: A-rich tails; Grey background: RNA pol III terminator.

**Table S1. PVY genome annotation.**

| **Name** | **Size** | **Annotation** |
| --- | --- | --- |
| 5′ UTR | 1–184 bp (184 bp) | 5′ untranslated region |
| P1 | 185–1009 (825 bp) | P1 protein |
| HC-Pro | 1010–2404 (1395 bp) | Helper component protease |
| P3^a^ | 2405–3499 (1095 bp) | P3 protein |
| 6K1 | 3500–3655 (156 bp) | 6K1 protein |
| CI^a^ | 3656–5557 (1901 bp) | Cylindrical or cytoplasmic inclusion |
| 6K2 | 5558–5713 (256 bp) | 6K2 protein |
| VPg | 5714–6277 (564 bp) | Viral genome-linked protein |
| NIa | 6278–7009 (732 bp) | First nuclear inclusion protein |
| NIb^a^ | 7010–8566 (1557 bp) | Second nuclear inclusion protein |
| CP^a^ | 8567–9367 (801 bp) | Coat protein |
| 3′ UTR | 9371–9698 (263 bp) | 3′ untranslated region |

^a^ The four different colors denote the four different targeting regions used in this study.

**Table S2. List of oligonucleotides used in this study.**

| **Oligonucleotide** | **Sequence 5′ to 3′** | **Description and Use** |
| --- | --- | --- |
| pP3-F | AAACGATGAAATATTTACACACTTAGTATTGA | Forward primer for cloning of crRNA-P3, introducing two BsaI sites |
| pP3-R | TTTTTCAATACTAAGTGTGTAAATATTTCATC | Reverse primer for cloning of crRNA-P3, introducing two BsaI sites |
| pCI-F | AAACAATGTCATGTATGACAGGATGCATTGAT | Forward primer for cloning of crRNA-CI, introducing two BsaI sites |
| pCI-R | TTTTATCAATGCATCCTGTCATACATGACATT | Reverse primer for cloning of crRNA-CI, introducing two BsaI sites |
| pCP-F | AAACTTGGTTTTGCATTCTCAACGATTGGTTT | Forward primer for cloning of crRNA-CP, introducing two BsaI sites |
| pCP-R | TTTTAAACCAATCGTTGAGAATGCAAAACCAA | Reverse primer for cloning of crRNA-CP introducing two BsaI sites |
| pNIb-F | AAACACCATGAGTAGAATCTCCTGATTTGGTG | Forward primer for cloning of crRNA-NIb, introducing two BsaI sites |
| pNIb-R | TTTTCACCAAATCAGGAGATTCTACTCATGGT | Reverse primer for cloning of crRNA-NIb, introducing two BsaI sites |
| sgRNA-F | GCCACCCCAATATCGAAGG | Forward primer for qRT-PCR analysis of sgRNA expression |
| P3-sgRNA-R | CTAAGTGTGTAAATATTTCATC | Reverse primer for qRT-PCR analysis of P3 expression |
| CI-sgRNA-R | ATCAATGCATCCTGTCATACAT | Reverse primer for qRT-PCR analysis of CI expression |
| CP-sgRNA-R | CCAATCGTTGAGAATGCAAAACC | Reverse primer for qRT-PCR analysis of CP expression |
| NIb-sgRNA-R | TCAGGAGATTCTACTCATGGTGT | Reverse primer for qRT-PCR analysis of NIb expression |
| St-Cas13a-F | CGGACTTCGAGAGCTGGGCA | Forward primer for qRT-PCR analysis of Cas13a expression |
| St-Cas13a-R | CGGCGAACGGGTTCCTAACGA | Reverse primer for qRT-PCR analysis of Cas13a expression |
| Tubulin-F | GATGTTGTGCCAAAGGATGT | Forward primer for qRT-PCR analysis of tubulin expression |
| Tubulin-R | AACTTGTGGTCAATGCGAGA | Reverse primer for qRT-PCR analysis of tubulin expression |
| PVY-3S | ACGTCCAAAATGAGAATGCC | Forward primer for qRT-PCR detection of PVY^O^, PVY^N^, and PVY^N:O^ |
| PVY-R1 | GCGAGGTTCCATTTTCAATGCA | Reverse primer for qRT-PCR detection of PVY^O^, PVY^N^, and PVY^N:O^ |
| PVY-FL-F | ACGTCCAAAATGAGAATGCC | Probe labelling for detection of PVY genome |
| PVY-FL-R | TGGTGT TCGTGATGTGACCT |
